# Antimicrobial Activities of Polysaccharide-Rich Extracts from the Irish Seaweed *Alaria esculenta*, Generated Using Green and Conventional Extraction Technologies, Against Foodborne Pathogens

**DOI:** 10.3390/md23010046

**Published:** 2025-01-18

**Authors:** Ailbhe McGurrin, Rahel Suchintita Das, Arturo B. Soro, Julie Maguire, Noelia Flórez Fernández, Herminia Dominguez, Maria Dolores Torres, Brijesh K. Tiwari, Marco Garcia-Vaquero

**Affiliations:** 1 Section of Food and Nutrition, School of Agriculture and Food Science, University College Dublin, Belfield, D04 V1W8 Dublin, Ireland; ailbhe.mcgurrin@ucdconnect.ie (A.M.); rahel.suchintitadas@ucdconnect.ie (R.S.D.); 2 TEAGASC, Food Research Centre, Ashtown, D15 DY05 Dublin, Ireland; brijesh.tiwari@teagasc.ie; 3 Departament de Nutrició, Ciències de l’Alimentació i Gastronomia, Facultat de Farmàcia i Ciències de l’Alimentació, Campus de l’Alimentació de Torribera, University of Barcelona, 08921 Barcelona, Spain; arturoblazquezsoro@ub.edu; 4 Institut de Recerca en Nutrició i Seguretat Alimentària (INSA·UB), University of Barcelona, 08921 Barcelona, Spain; 5 Bantry Marine Research Station Ltd., Gearhies, Bantry, P75 AX07 Co. Cork, Ireland; jmaguire@bmrs.ie; 6 Grupo de Biomasa y Desarrollo Sostenible, Departamento de Ingeniería Química, Facultad de Ciencias, Universidade de Vigo, 32004 Ourense, Spain; noelia.florez@uvigo.gal (N.F.F.); herminia@uvigo.gal (H.D.); matorres@uvigo.gal (M.D.T.)

**Keywords:** bioactive, antimicrobial, *Alaria esculenta*, polysaccharide, green chemistry, bio-preservative, seaweed

## Abstract

A rise in antimicrobial resistance coupled with consumer preferences towards natural preservatives has resulted in increased research towards investigating antimicrobial compounds from natural sources such as macroalgae (seaweeds), which contain antioxidant, antimicrobial, and anticancer compounds. This study investigates the antimicrobial activity of compounds produced by the Irish seaweed *Alaria esculenta* against *Escherichia coli* and *Listeria innocua*, bacterial species which are relevant for food safety. Microwave-assisted extraction (MAE), ultrasound-assisted extraction (UAE), ultrasound–microwave-assisted extraction (UMAE), and conventional extraction technologies (maceration) were applied to generate extracts from *A. esculenta*, followed by their preliminary chemical composition (total phenolic content, total protein content, total soluble sugars) and antimicrobial activity (with minimum inhibitory concentration determined by broth microdilution methods), examining also the molecular weight distribution (via high performance size exclusion chromatography) and oligosaccharide fraction composition (via high-performance liquid chromatography) of the polysaccharides, as they were the predominant compounds in these extracts, aiming to elucidate structure–function relationships. The chemical composition of the extracts demonstrated that they were high in total soluble sugars, with the highest total sugars being seen from the extract prepared with UAE, having 32.68 mg glucose equivalents/100 mg dried extract. Extracts had antimicrobial activity against *E. coli* and featured minimum inhibitory concentration (MIC) values of 6.25 mg/mL (in the case of the extract prepared with UAE) and 12.5 mg/mL (in the case of the extracts prepared with MAE, UMAE, and conventional maceration). No antimicrobial activity was seen by any extracts against *L. innocua*. An analysis of molar mass distribution of *A. esculenta* extracts showed high heterogeneity, with high-molecular-weight areas possibly indicating the presence of fucoidan. The FTIR spectra also indicated the presence of fucoidan as well as alginate, both of which are commonly found in brown seaweeds. These results indicate the potential of antimicrobials from seaweeds extracted using green technologies.

## 1. Introduction

Antimicrobial resistance is becoming an increasingly concerning issue for human health.The World Health Organisation declared antimicrobial resistance one of the top ten global public health threats, and the landmark 2014 report by economist Jim O’Neill predicts that by 2050 over 10 million global deaths each year will be a result of infection from antimicrobial-resistant microorganisms [1,2]. Moreover, there is a global paucity of new conventional antibiotics in the pipeline which has caused a shift in research towards antibiotics from alternative sources. In recent years, antimicrobial compounds from natural sources, such as those from terrestrial plants and marine organisms, have gained the attention of the research community as a potential resource to obtain novel antimicrobials from relatively unexplored forms of biomass. These novel antimicrobials may be able to act in concert with conventional antibiotics or act as a replacement for antibiotics if their own antimicrobial activity is robust enough.

Macroalgae (seaweeds) have emerged as one such biomass with reported antimicrobial activities [3,4,5]. The marine environment can often pose extreme conditions, including large variations in abiotic factors such as pH, UV irradiation, and oxygen availability, and biotic factors, such as microbial competitors, and pathogens [6]. Macroalgae produce a wide variety of secondary metabolites, such as polysaccharides, phenolic compounds, lipids, and terpenoids, which act as defence mechanisms against these harsh environmental factors [7,8]. Many of these secondary metabolites from macroalgae have been recently investigated as bioactive compounds or compounds that can be beneficial to human health. Multiple biological activities have been reported from compounds isolated from macroalgae including antioxidant, anti-inflammatory, antimicrobial, anticancer, and anti-hypertensive [3,9,10]. In an ecological context, antimicrobial compounds produced by macroalgae contribute towards their defence against biofouling organisms and pathogens. These antimicrobial compounds may also be useful for pharmaceutical and biotechnological applications. Antimicrobial activities of polysaccharides from macroalgae have been increasingly reported in recent years. Fucoidan and laminarin from brown macroalgae have been described as carbohydrates with strong antimicrobial activity against a wide variety of bacteria [11,12,13].

Concurrent with the rise in searching for novel antimicrobials from natural sources is the rise in consumer demand for food preservatives derived from natural origins. Ensuring food products do not become contaminated by microorganisms is a cornerstone of food preservative treatments, with the most common foodborne pathogens being represented by *Escherichia coli* and *Listeria* spp. amongst others. Conventionally, food preservatives comprise chemically synthesised agents, including nitrates/nitrites and the antioxidants BHA and BHT [14]. In recent years, consumer preferences have moved away from chemically derived food preservatives; nitrates/nitrites have been linked with carcinogenic properties [15], and, with the increasing number of climate crises, more consumers have shown a preference for agriculture and food processing which does not rely on chemically synthesised compounds. Natural antimicrobial compounds from terrestrial plants, such as herbs, spices, Cruciferae and hops, have already been investigated as potential bio-preservatives, that is, food preservatives derived from natural origin [3,16]. Thus, bioactive compounds from macroalgae with antimicrobial properties could pose a potential solution to the dual global challenges of lack of novel antimicrobials in the pipeline, and shift in consumer preferences towards bio-preservatives.

Research on bioactive compounds from natural sources has increasingly shifted towards sustainable methods of extracting these compounds from inside the biomass to be studied. Conventional extraction methods often involve temperatures >100 °C, environmentally damaging solvents, and long extraction times [9,17]. Next-generation extraction technologies such as ultrasound-assisted extraction (UAE) [18], microwave-assisted extraction (MAE) [19], and supercritical fluid extraction (SFE) [20] have the ability to preserve the extraction efficiency of bioactive compounds while utilising greener solvents, lower temperatures, and reduced extraction times [5,21,22]. Different extraction technologies can influence the nature and activity of bioactive compounds isolated from seaweeds; Dang, Bowyer [23] reported that MAE resulted in higher phenolic content extractions than UAE, while Garcia-Vaquero, Ravindran [22] reported enhanced phytochemical extraction due to UAE over MAE. Depending on the particular classes of chemical compounds extracted by different technologies (phenolic compounds, polysaccharides, lipids), the associated bioactivities of these compounds will also differ.

*Alaria esculenta* (winged kelp) is an edible brown macroalgae found in temperate waters of the North Atlantic Ocean, including in Ireland, Scotland, and Greenland [24]. High in minerals and vitamins, is it commercially available as a food product or used in animal feed. Bioactive properties, such as antioxidant- and Cu^2+^-chelating activities, have been previously reported from this species [24,25,26], as well as high contents of polyunsaturated fatty acids (PUFAs), which are beneficial for cardiovascular health [27]. While there are emerging studies analysing the antimicrobial activities of extracts of *A. esculenta* [24,28], to the best of the authors’ knowledge, its antibacterial activity against the food safety pathogen *E. coli* has not yet been reported.

This study assesses the antimicrobial activity against *E. coli* and *Listeria innocua* of polysaccharide-rich extracts from the Irish brown macroalga *A. esculenta* achieved by green extraction technologies and procedures, by MAE, UAE, and a combination of ultrasound–microwave-assisted extraction (UMAE), and conventional extraction technologies, such as maceration. The molecular weight distribution and oligosaccharide fraction composition of the extracts were also determined, aiming to elucidate the structure–function relationships of these bioactives.

## 2. Results

### 2.1. Chemical Composition

**Figure 1:**
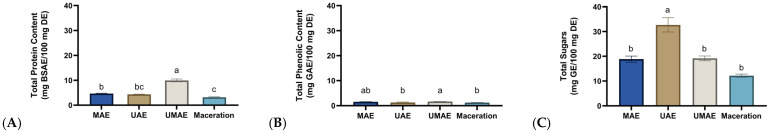
Summary of the (**A**) total protein content, (**B**) total phenolic content, and (**C**) total sugar content of extracts of *A. esculenta* prepared by either microwave-assisted extraction (MAE), ultrasound-assisted extraction (UAE), ultrasound–microwave-assisted extraction (UMAE), or maceration, as reported by [29]. Results are expressed as the average ± standard error of the mean (SEM) with n = 6. Different letters on bars indicate a significant difference (*p* < 0.05) between means. Abbreviations within the figure are as follows: BSAEs (bovine serum albumin equivalents), GAEs (gallic acid equivalents), GEs (glucose equivalents), and DEs (dried extract).

The chemical composition of the *A. esculenta* extracts selected for this study were determined in a previous study [29] and are summarised in Figure 1. The extracts selected had high levels of total soluble sugars (TSS), with UAE having the highest TSS levels. In contrast, all the extracts had, in general, low levels of TPC and total proteins ranging between 1.27 and 1.65 mg gallic acid equivalents/100 mg dried extract and between 3.15 and 9.93 mg bovine serum albumin equivalents/100 mg dried extract, respectively.

### 2.2. Antimicrobial Analyses

#### 2.2.1. Minimum Inhibitory Concentration (MIC) Assay

**Figure 2:**
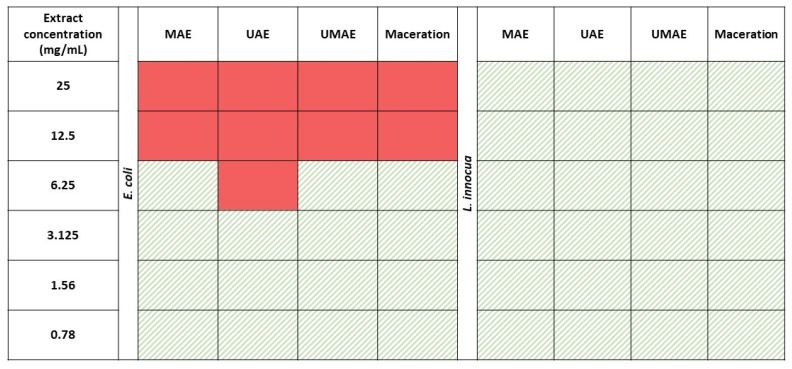
Antimicrobial activity of extracts of *A. esculenta* at concentrations of 0.78–25 mg/mL prepared via either ultrasound-assisted extraction (UAE), microwave-assisted extraction (MAE), a combination of ultrasound–microwave-assisted extraction (UMAE), or maceration extracts against *E. coli* and *L. innocua*. A solid-red colour indicates inhibition of bacterial growth, while green patterned cells indicate bacterial growth.

The results of the MIC assays are depicted in Figure 2. In the case of *E. coli*, the extract prepared with MAE inhibited bacterial growth at concentrations ≥ 12.5 mg/mL (MIC), the extract prepared with UAE had a MIC of 6.25 mg/mL, the extract prepared with UMAE had a MIC of 12.5 mg/mL, and the extract prepared using conventional maceration had an MIC of 12.5 mg/mL. Extracts of *A. esculenta* did not show any antimicrobial activity towards *L. innocua*, even at the highest concentrations tested (25 mg/mL).

#### 2.2.2. Bacterial Growth Curves

**Figure 3:**
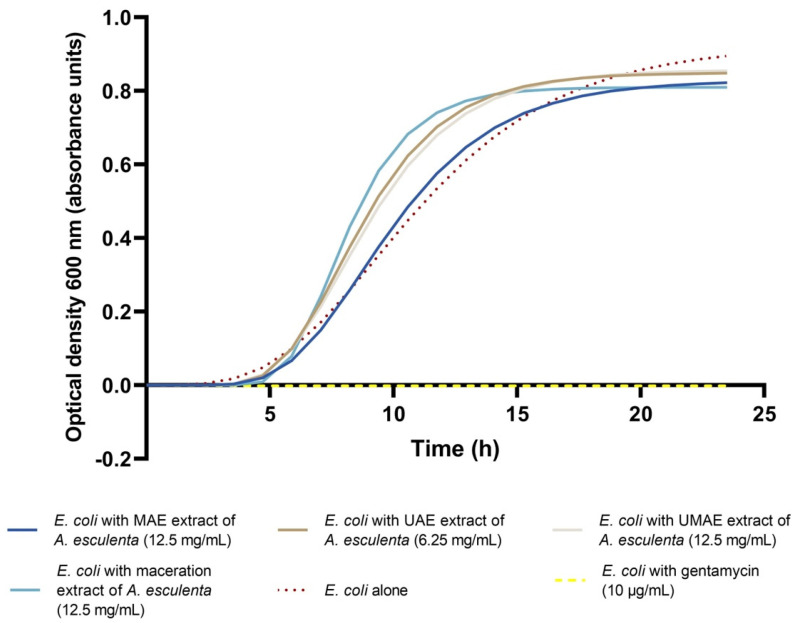
Gompertz modelling of *E. coli* incubated with *A. esculenta* extracts prepared via either microwave-assisted extraction (MAE), ultrasound-assisted extraction (UAE), a combination of ultrasound–microwave-assisted extraction (UMAE), or maceration extraction over 24 h (each line portrays a representative sample). All extracts were applied at their MIC, determined above. A control of *E. coli* incubated with an antibiotic (gentamycin at 10 µg/mL) was also included.

After initial MIC determination, extracts of *A. esculenta* prepared with either MAE, UAE, UMAE, or maceration were incubated with *E. coli* for 24 h and the optical density was measured every 30 min. The results of this growth curve were subjected to Gompertz modelling, which is detailed in Figure 3. Extrapolation of Gompertz modelling yielded the growth parameter rate (μ_max_) and lag (λ), as summarised in Table 1. The optical density of *E. coli* alone, with no inhibitory compounds, was recorded as a control. In the presence of extracts of *A. esculenta* at their MIC prepared via either UAE, MAE, UMAE, or maceration, the rate of microbial growth (μ_max_) did not decrease in comparison with the control. It was observed that the rate of growth of *E. coli* increased significantly in the presence of *A. esculenta*. In contrast, regarding the lag phase (λ) for *E. coli*, it was observed that the addition of the *A. esculenta* extract prepared with MAE extended the length of the lag phase significantly in comparison with the control, where the lag phase of *E. coli* was extended to 5.73 h. The addition of *A. esculenta* that was prepared via UAE, UMAE, and by maceration did not have a significant effect on the lag phase of *E. coli* growth.

### 2.3. Polysaccharide Analyses

#### 2.3.1. Molecular Weight Distribution

**Figure 4:**
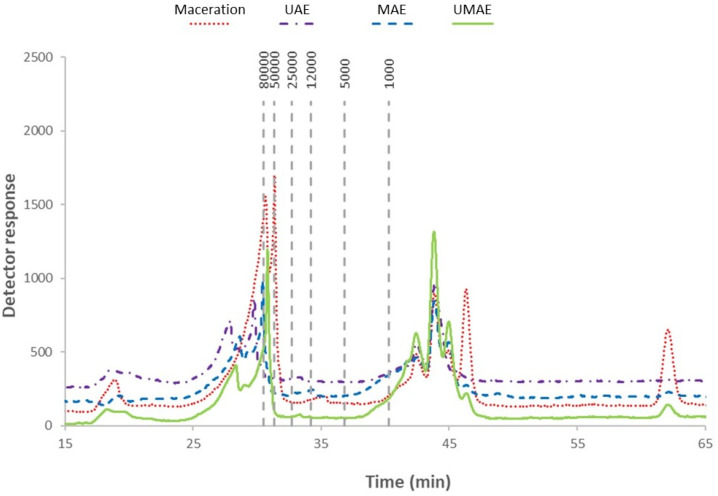
Molecular weight distribution profile of extracts of *A. esculenta* prepared with either microwave-assisted extraction (MAE), ultrasound-assisted extraction (UAE), ultrasound–microwave-assisted extraction (UMAE), or maceration. Marks within the figure represent molecular weight (80,000–1000) in Daltons of the dextran standard.

The molecular weight profile of extracts of *A. esculenta* is depicted in Figure 4. Two well-defined areas were observed that were associated with low molecular weights (for polymer size < 10 kDa) and medium molecular weights (for polymers between 10 and 10,000 kDa) [30]. Peaks with retention times longer than 45 min were found in the extract achieved by maceration, eluted after the standard dextran of 1000 Da, indicating the presence of compounds with smaller molecular weights. All samples exhibited peaks above 50 kDa, with higher intensities being found for extracts prepared by UMAE and maceration.

#### 2.3.2. FTIR

**Figure 5:**
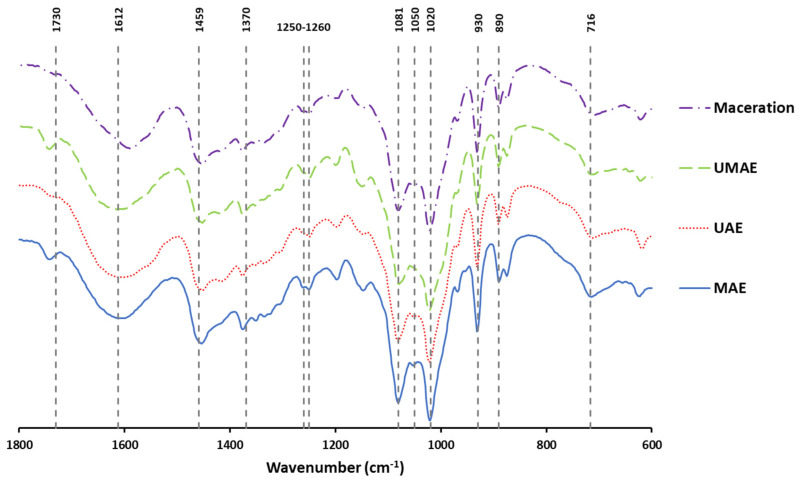
FTIR spectra of extracts obtained from *A. esculenta* using either microwave-assisted extraction (MAE), ultrasound-assisted extraction (UAE), ultrasound–microwave-assisted extraction (UMAE), or maceration.

The FTIR spectra of the extracts are exhibited in Figure 5, where the wave number region studied was from 1800 cm^−1^ to 600 cm^−1^.

#### 2.3.3. Oligosaccharide Composition

After hydrolysis, the oligosaccharide composition of extracts from *A. esculenta* were analysed and summarised in Table 2. The extract prepared with UMAE had the largest amounts of oligosaccharides (≈50%), followed by the maceration extract (46%).

### 2.4. Pearson’s Correlation Matrix and Principal Component Analysis (PCA)

**Figure 6:**
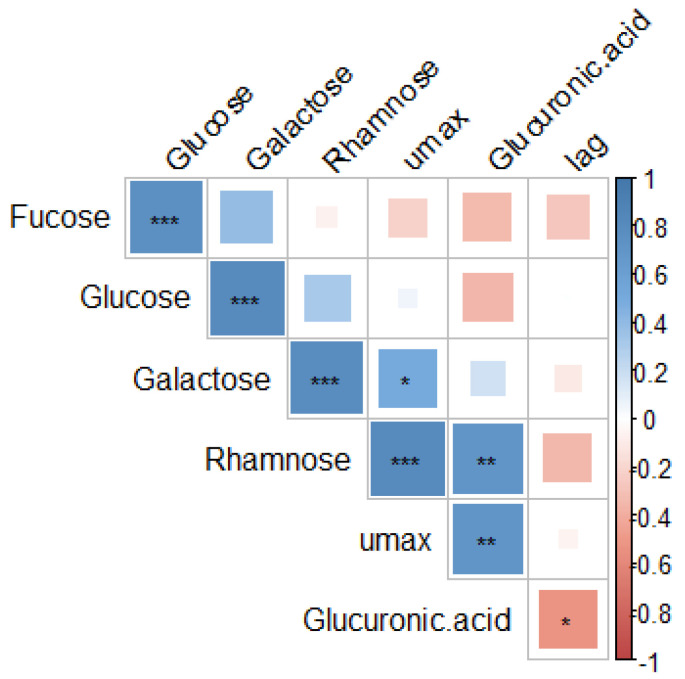
Correlation matrix of the parameters of microbial growth (rate (µ_max_) and lag (λ)) and the oligosaccharide fraction composition of extracts of *A. esculenta*. Abbreviations in the figure are as follows: µ_max_ (rate), λ (lag). The statistical significance of the correlations is indicated in the figure as * *p* < 0.05, ** *p* < 0.01, *** *p* < 0.001.

A correlation matrix (Figure 6) was carried out to examine the relationship between oligosaccharide fractions present in extracts of *A. esculenta* and the effects of *A. esculenta* extracts on microbial growth parameters. The increased microbial growth rate (µ_max_) was correlated with the presence of rhamnose and glucuronic acid (*p* < 0.05). Increased microbial lag time (λ) was associated with glucuronic acid (*p* < 0.05).

**Figure 7:**
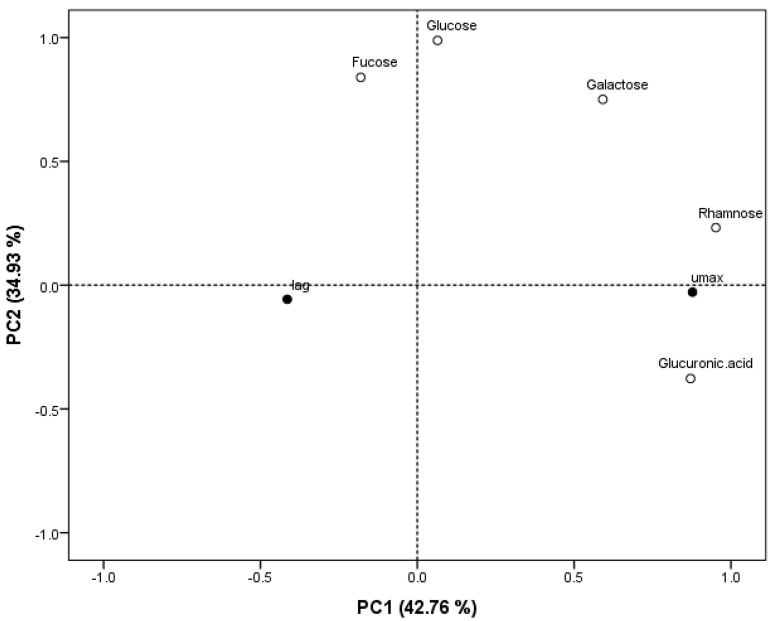
Principal Component Analysis (PCA) scatter plot representing the scores for parameters of microbial growth (rate (µ_max_) and lag (λ)) and oligosaccharide fraction composition of extracts of *A. esculenta*. Abbreviations in the figure are as follows: µ_max_ (rate), λ (lag).

A Principal Component Analysis (PCA) (Figure 7) was performed to analyse the similarities and differences between the oligosaccharide fraction composition of *A. esculenta* extracts with respect to the growth parameter rate (µ_max_) and lag (λ) that were determined in the antimicrobial analysis in this study. Together, the two PCs (principal components) obtained here explain 77.69% of the cumulative variation in the data, with PC1 accounting for 42.76% of the data variation and PC2 accounting for 34.93%.

## 3. Discussion

### 3.1. Chemical Composition

In this study, treatment with UAE extracted significantly more TSS than either MAE or UMAE. Macroalgal polysaccharides are often contained within the biomass cell wall, having a structural function, and thus, these compounds can be difficult to extract from the biomass [31]. The application of UAE on the biomass results in a variety of physical and chemical phenomena, including shear forces and cavitation, resulting in compression, rarefaction, and acoustic streaming [18]. Previous work reported an enhanced phytochemical extraction due to UAE over MAE [22], which may be the case here. Interestingly, Schiener, Black [32] reported similar comparative chemical composition results from brown macroalgae, including *A. esculenta*, whereby the extracts with the highest content of polysaccharides, such as laminarin and mannitol, were the lowest in polyphenols (as well as the lowest in protein, ash, and moisture). The authors suggested that this result could be used to guide extraction of brown macroalgae to target extraction of certain chemical compounds.

The antimicrobial activity of macroalgal polysaccharides has been reported previously [11,13,33], particularly from sulfated polysaccharides, such as fucoidan, galactan, and ulvan, and non-sulfated polysaccharides, such as laminarin [34]. The antimicrobial mechanism of action of polysaccharides has been linked to interactions with the bacterial cell wall, resulting in membrane leakage, as well as interactions of negatively charged sulfated polysaccharides with surrounding cationic nutrients in the bacterial medium [33]. However, the antimicrobial activity of these polysaccharides can be influenced by the relative proportion of different polysaccharides within polysaccharide-rich extracts [33,35], as well as differences in the structure of these compounds, including molecular weight and monosaccharide composition [36,37]. A wide variety of factors can influence the abundance of macroalgal polysaccharides, their chemical structure, and, thus, their biological properties, including seasonal and environmental conditions affecting the biomass (for example, [32] reported higher levels of laminarin in the autumn and lower levels in the winter) as well as the extraction technology and conditions used to generate polysaccharide-rich ingredients (i.e., parameters such as temperature, pH, pressure, extraction time, extraction solvent) [38,39].

### 3.2. Antimicrobial Analyses

Few studies focus on the biological activities of polysaccharides or polysaccharide-rich extracts from *A. esculenta*, with the majority of studies being focused mainly on alginates and cellulose for industrial applications [40,41] and fucoidan and laminarin. Birgersson, Oftebro [42] sequentially extracted fucoidan and laminarin from *A. esculenta*, but the biological activities of these compounds were not analysed in this study. Similarly, Rhein-Knudsen, Reyes-Weiss, and Horn [43] extracted high-purity fucoidans from *A. esculenta*, but their biological activities were not analysed. Zhu, Healy [28] did characterise the biological activity of laminarin extracted from *A. esculenta* using hydrodynamic cavitation and reported antioxidant, anti-inflammatory, and antimicrobial activity against *Bacillus subtilis*. In the present study, *A. esculenta* extracts contained a greater quantity of TSS than any other chemical components analysed, and thus the authors suggest that the antimicrobial activity of these extracts is related to their polysaccharide-rich nature. It should be noted that crude extracts contain complex mixes of various chemical compounds and small molecules [44,45]. For the scope of this study, only the polysaccharide content of the extracts was further explored. The extent to which other chemical components within the extracts interact with each other to form synergistic/antagonistic antimicrobial activities is unknown. The extract of *A. esculenta* prepared with UAE had the strongest antimicrobial activity against *E. coli* with the lowest numerical MIC of the extracts tested. This extract also had the highest level of TSSs, and it was significantly greater than the other extracts. Thus, the enhanced antimicrobial effect from this extract could be attributed to the abundance of total sugars present in this extract. Antimicrobial activity from extracts of macroalgae have been reported on in numerous studies [46,47,48]. Polysaccharides have been identified as one of the main classes of bioactive compounds within macroalgae that possess antimicrobial activity [11,13], with the main studies being represented by sulfated polysaccharides [49]. The degree of antimicrobial activity of isolated polysaccharides or polysaccharide-rich extracts from macroalgae can vary greatly depending on the species of microorganism targeted and the methods implemented [50].

The antimicrobial activity of *A. esculenta* (crude extracts or isolated compounds) remains understudied. Most studies investigating biological activity from *A. esculenta* extracts have focused on antioxidant [24], ACE-inhibiting, and Fe^2+^ or Cu^2+^ activity [27]. Sapatinha, Oliveira [24] tested crude extracts from four seaweed species, including *A. esculenta*, for antimicrobial activity against *Citrobacter freundii*, *Enterococcus faecalis*, *E. coli*, *L. monocytogenes*, *Pseudomonas aeruginosa*, *Salmonella* Typhimurium, and *Staphylococcus aureus*. No antimicrobial activity was reported from *A. esculenta* extracts nor the other seaweed species tested. Zhu, Healy [28] reported antimicrobial activity from laminarin extracted from *A. esculenta*, where laminarin reduced the rate of growth of *B. subtilis*. This study also tested the activity of the laminarin against *E. coli* and *Saccharomyces cerevisiae* and found no antimicrobial activity. Thus, as far as the authors are aware, the present study is the first report of antimicrobial activity by polysaccharides from *A. esculenta* against the important food pathogen *E. coli*.

The current study reports MIC values of 6–12.5 mg/mL from this polysaccharide-rich extract of *A. esculenta*, which is in a similar order of magnitude to MIC values reported in certain other studies. Rajauria, Jaiswal [51] reported an MIC of 60 mg/mL from a crude extract of *Himanthalia elongata* against a range of bacteria. Otero, Quintana [52] observed an IC_50_ of 2.24 mg/mL against *E. coli*, and Liu, Liu [33] reported an MIC of 6 mg/mL against *E. coli* from a depolymerised fucoidan fraction. Other studies showed more potent antimicrobial activity from macroalgal extracts: Nshimiyumukiza, Kang [53] reported an MIC of 256 µg/mL from an extract of the brown seaweed *Ecklonia cava*, Martins, Nedel [10] reported an MIC of 500 µg/mL against *E. faecalis* from an extract of *Cystosphaera jacquinotii*, and Palanisamy, Vinosha [13] studied a crude fucoidan fraction which had an MIC of 200 µg/mL against *E. coli*. Generally, large variations can be seen in the results of the antimicrobial activity of macroalgal extracts, with certain studies reporting extensive antimicrobial activity against multiple microorganisms [37,54] while others report antimicrobial activity towards some microorganisms but not others [55]. The extent of antimicrobial activity from macroalgal polysaccharides, and macroalgal extracts in general, is influenced by a variety of factors, such as chemical structures and conformation, molecular weight, charge density, and sulphate content in the case of sulphated polysaccharides [4]. While certain studies have reported broad spectrum antimicrobial activity from macroalgal extracts and macroalgal polysaccharides [56], others have shown targeted effects on either Gram-negative or Gram-positive bacteria. The current study observed antimicrobial activity against the Gram-negative *E. coli* only, with no activity against the Gram-positive *Listeria innocua*. Amorim, Rodrigues [57] also reported antimicrobial activity from macroalgal polysaccharides against *E. coli* but not against certain Gram-positive bacteria tested (*B. subtilis*, *S. aureus*). However, this study also reported no antimicrobial activity against other Gram-negative bacteria, including *P. aeruginosa*. Interestingly, Yamashita, Yoshiko, and Shimizu [58] reported antimicrobial activity from macroalgal carrageenan against *E. coli* and other Gram-negative and Gram-positive bacteria but not *Listeria* spp. Other studies found no antimicrobial effects on Gram-negative bacteria by macroalgal extracts [12]. Overall, the results of this study indicate promising antimicrobial activity for the brown seaweed *A. esculenta* against *E. coli*, a relevant pathogen for food safety.

As was observed in the bacterial growth curves in this study, previous studies also observed an increase in microbial biomass after the addition of antimicrobial extracts of a natural origin. Carneiro, Dos Santos [59] studied the antimicrobial activity of casbane diterpene (CD) isolated from a plant extract and reported an increase in *P. aeruginosa* biomass upon addition of the CD at sub-inhibitory levels. The authors suggested that this may be due to an increased production of exopolysaccharides by *P. aeruginosa* as a response to treatment of CD. Cabral, Mondala [37] reported a similar result with increased microbial biomass after treatment with a fucoidan-rich extract from *Fucus vesiculosus*. Furthermore, Jun, Jung [12] investigated the antimicrobial activity of sulphated polysaccharides from macroalgae and reported that fucoidan isolated from *F. vesiculosus did* not possess a direct killing effect for microbial cells. Rather, the authors reported an indirect antimicrobial effect which they suggest was caused by the ability of fucoidan to trap nutrients in the medium. Future research should explore microbe–antimicrobial agent interactions at a molecular level to better understand mechanisms of action beyond direct killing effects. The extension of the lag phase of *E. coli* by extracts of *A. esculenta* indicates the bacteriostatic nature of these extracts and opens avenues of further development of these extracts in the food industry as food preservatives, a household cleaning agent, or as a pharmaceutical antibacterial agent.

### 3.3. Polysaccharide Analysis

In the molecular weight distribution profile of *A. esculenta* extracts, a low-molecular-weight area (<10 kDa) and a medium-molecular-weight area (between 10 and 10,000 kDa) were defined. Gómez-Ordóñez, Jiménez-Escrig, and Rupérez [60] also reported the high heterogeneity of the molecular weight of polysaccharides present in the brown seaweed *Saccharina latissima.* High performance size exclusion chromatography (HPSEC) yielded four defined peaks associated with 5.7–5.8 kDa (which the authors tentatively identified as laminarin), 23–27 kDa, 338–351 kDa, and 2111–2190 kDa. The authors attributed the heterogeneity of polysaccharides in *S. latissima* to the complex polysaccharide composition of brown macroalgae as well as the extraction process and time of collection. The differences in molecular weight profiles observed here could be due to the different extraction technologies applied. For example, the lower-molecular-weight peaks observed in the maceration extract could represent heat-sensitive compounds which were degraded in the other treatments where either ultrasound and/or microwaves were applied. Tsubaki, Oono [61] reported that conditions of microwave extraction can impact polysaccharide molecular weight and viscosity in *Ulva* spp., while Rodriguez-Jasso, Mussatto [62] noted that fucoidan yields from *F. vesiculosus* were improved by increasing pressure within the microwave extraction system. Laminarin has a low molecular weight of around 4–5 kDa [63], which may be representative of the low-molecular-weight band observed in this study. The medium-molecular-weight band (between 10 and 10,000 kDa) may be representative of fucoidans, as this group of compounds are often characterised by high molecular weights reaching 950 kDa [64] but which can span a variety of molecular weights depending on specific extraction conditions [65]. Fucoidans belong to the family fucose-rich sulphated polysaccharides, compounds of heterogenous polymeric structure of sulphated fucose or other monosaccharides [66]. Among other bioactivities (antioxidant, anticancer) [65,67], the antimicrobial activity of fucoidans has been reported [12,68,69]. Palanisamy, Vinosha [13] reported the antibacterial activity of a fucoidan fraction isolated from *Sargassum polycystum* against *E. coli*, with an MIC of 200 mg/mL. The same study also reported antibacterial activity against *P. aeruginosa*, *Streptococcus mutans*, and *S. aureus.* Liu, Liu [33] reported the antibacterial activity of fucoidan against *E. coli*, where a depolymerised fucoidan isolated from *Saccharina japonica* (formerly *Laminaria japonica*) inhibited *E. coli* and *S. aureus*. This study by Liu, Liu [33] reported stronger antibacterial activity against a Gram-negative organism (*E. coli*) than a Gram-positive organism (*S. aureus*), similar to what is being reported here in this study, where antibacterial activity is observed against *E. coli* but not *L. innocua*. Cabral, Mondala [37] observed antibacterial activity against *E. coli* from a fucoidan-rich extract from the brown seaweed *F. vesiculosus*, as well as antibacterial activity against pathogens related to food safety such as *B. subtilis*, *P. aeruginosa*, and *L. innocua*.

In the FTIR spectra, the band obtained at 716 cm^−1^ was associated with C-O-C bending vibrations and glycosidic linkages, and the peak obtained at 890 cm^−1^ was assigned to anomeric C-H of β-galactopyranosyl residues [70]. The peak obtained at 930 cm^−1^ was reported as uronic acid residues (C-O stretching vibration) [71]. The bands observed at 1020 and 1081 cm^−1^ can be related to stretching vibrations of the pyranose ring (C-O and C-C), and a slight peak at 1050 cm^−1^ (observed in the extract prepared with MAE and maceration extracts) was attributed to a stretching vibration of C-O-C [72]. The bands observed at 1250 cm^−1^ and 1260 cm^−1^ can be attributed to the presence of asymmetric O=S=O stretching vibrations of a sulphate group and to the stretching vibration of the S-O of sulphate, respectively [73], that are indicative of the presence of fucoidan [74]. The peak observed at 1370 cm^−1^ is related to the -C-O=S stretching vibration and to the -C-O-S group [74]. The bands at 1459 and 1612 cm^−1^ could be associated with carboxyl groups [75]. The small signal obtained at 1730 cm^−1^ is characteristic of alginate and is associated with carboxylic acid in ester form (C=O) [71]. Fucoidan and alginate are some of the most common polysaccharides reported from brown macroalgae [74], with a variety of bioactivities being associated with these compounds (antioxidant, anti-inflammatory, antimicrobial).

An oligosaccharide composition analysis showed that the extract prepared with UMAE had the largest amounts of oligosaccharide followed by the maceration extract. A similar value of the total quantity of these compounds was also achieved by Cebrián-Lloret, Metz [76]. In both cases, glucose was the main saccharide (highest for the extract prepared with UMAE), and similar values were obtained using acidic extraction [77]. This behaviour is associated with the extraction process [78,79]. Brown macroalgae, such as *A. esculenta*, have been reported to have glucose as the main oligomer, with the major reported saccharides being laminarin and fucoidan [80,81].

### 3.4. Pearson’s Correlation Matrix and Principal Component Analysis (PCA)

A correlation matrix was carried out to examine the relationship between oligosaccharide fractions present in extracts of *A. esculenta* and the effects of *A. esculenta* extracts on microbial growth parameters. Increased microbial growth rate (µ_max_) was correlated with the presence of rhamnose and glucuronic acid (*p* < 0.05). Increased microbial lag time (λ) was associated with glucuronic acid (*p* < 0.05), which may be related to the antimicrobial effect of these extracts on *E. coli.* Glucuronic acid is one of the main components of ulvans, polysaccharides from green seaweeds which have reported antimicrobial activity [82,83,84].

Principal Component Analysis (PCA) was performed to analyse the similarities and differences between the oligosaccharide fraction composition of *A. esculenta* extracts with respect to the growth parameter rate (µ_max_) and lag (λ), that were determined in the antimicrobial analysis in this study. PC1 appears to cluster the microbial growth rate (µ_max_) with the presence of the oligosaccharide fractions glucuronic acid, rhamnose, galactose, and glucose, while the microbial lag (λ) appears to be clustered with the presence of fucose. PC2 further explains the variation in data by appearing to relate fucose, glucose, galactose and rhamnose to each other, while µ_max_ and λ appear to be related with the presence of glucuronic acid. A correlation of lag (λ) with fucose indicates that the presence of fucose may be related to increased lag times of microbial growth, and thus fucose may be representative of the antimicrobial effects of *A. esculenta* extracts against *E. coli.* This is supported by numerous reports of the antimicrobial activity observed from fucose-containing polysaccharides from macroalgae, or fucoidans, in macroalgae [4,21,33]. Future studies should aim to further purify extracts of *A. esculenta* that show antimicrobial activity to further elucidate the bioactivities of this species.

## 4. Materials and Methods

### 4.1. Extract Preparation and Chemical Composition

**Figure 8:**
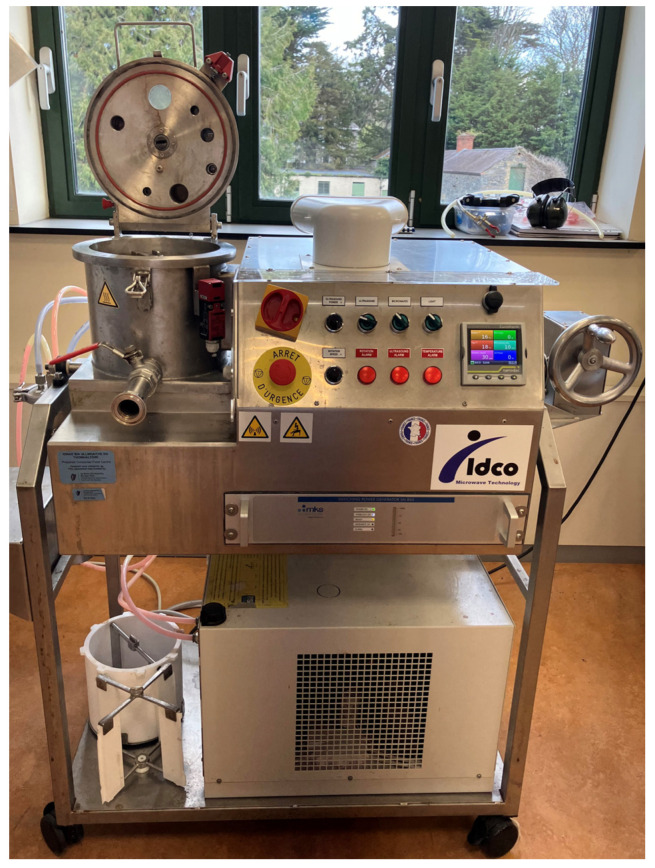
E200 Ultrasound–Microwave extractor (IDCO, Marseille, France).

Extracts explored in this study were generated in a previous study [29]. Fresh *A. esculenta* was supplied by Dúlra Ltd. and harvested from Blacksod Bay, Mayo, Ireland, in April 2021. Extracts of *A. esculenta* were prepared using MAE, UAE, or UMAE using an IDCO E200 extractor (IDCO SAS, Marseille, France) (Figure 8 below) of 1.25 L capacity, which allows for UAE, MAE, or simultaneous UMAE. Fresh seaweed samples were extracted using distilled water at a seaweed–solvent ratio 1:10 *w*/*v* and extracted by multiple sessions of MAE, UAE, UMAE, and maceration using multiple conditions. The mixtures were filtered and the extracts were freeze-dried and stored in vacuum-packed containers at −20 °C for further analyses. Following the determination of their chemical composition through total soluble sugars [85], total phenolic content [86], and total protein [87], the extracts achieved by UAE (200 W ultrasound power for 20 min), MAE (1340 W microwave power for 20 min), UMAE (200 W ultrasound power and 1340 W microwave power for 20 min), and control (maceration for 20 min) were selected for this antimicrobial study on the basis of their high content in polysaccharides (chemical composition results above in Figure 1).

### 4.2. Antimicrobial Analyses

#### 4.2.1. Microorganisms

The antimicrobial activity of *A. esculenta* extracts was tested using *E. coli* DSM1103 and *L. innocua* NCTC 11288. These two bacterial species were selected to model the pathogenic bacteria (*E. coli* and *L. monocytogenes*) due to their biological similarity [88,89]. Bacterial strains were obtained from Teagasc National Food Research Centre (Dublin, Ireland) and were maintained at −80 °C in 25% glycerol. Each bacterial strain was streaked onto Tryptone Soy Agar (TSA, Oxoid, Basingstoke, UK) and incubated at 37 °C for 24 h in aerobic conditions. Then, a single colony was used to inoculate 10 mL Tryptone Soy Broth (TSB, Oxoid, UK), which was incubated at 37 °C for 20 h in aerobic conditions.

#### 4.2.2. Minimum Inhibitory Concentration (MIC) Assay

The minimum inhibitory concentrations of the extracts of *A. esculenta* were determined according to an established protocol [90]. Assays were performed in 96-well microtitre plates. Briefly, bacteria were grown to a mid-exponential phase in overnight cultures, diluted in Maximum Recovery Diluent (MRD, Oxoid, UK) and 50 µL used to inoculate microtitre plates. The concentration of bacteria used was confirmed by enumerating serial dilutions of bacteria which were plated on TSA and incubated at 37 °C for 24 h in aerobic conditions. Extracts of *A. esculenta* were diluted in sterile water, and 50 µL were added to the microtitre plate wells to achieve the following concentrations of extract to be tested: 25, 12.5, 6.25, 3.12, 1.56, and 0.78 mg/mL. An amount of 100 µL Muller-Hinton Broth (MHB, Oxoid, UK) was added to all wells. Wells containing MHB alone, bacteria incubated with MHB only, and bacteria incubated with gentamycin (10 µg/mL) were included as controls. The microtitre plates were covered and incubated at 37 °C for 24 h. Then, 40 µL of iodonitrotetrazolium chloride (INT, Sigma-Aldrich, St. Louis, MO, USA) at 0.2 mg/mL was added to each well as an indicator of microbial growth [91], which turns a pink colour in the presence of microbial growth. The MIC was determined as the lowest concentration of each extract where no pink colour was observed after incubation of 2 h with INT dye. All measurements were carried out in triplicate on independent days with at least three replicates per experiment.

#### 4.2.3. Bacterial Growth Curve Analysis

The antimicrobial effects of extracts of *A. esculenta* over time were analysed via a bacterial growth curve analysis using 96-well microtiter plates. Briefly, 50 µL extracts of *A. esculenta* at their MIC were incubated with 50 µL of either *E. coli* or *L. innocua* which was grown to a mid-exponential phase, as above. An amount of 100 µL MHB were added to each well. Wells containing MHB alone, bacteria incubated with MHB only, and bacteria incubated with gentamycin (10 µg/mL) were included as controls. Microtitre plates were covered and incubated at 37 °C for 24 h in a plate reader (Tecan, Männedorf, Switzerland), with optical density (OD) measurements at 600 nm being conducted every 30 min. All measurements were performed in triplicate on independent days with at least three replicates per experiment.

### 4.3. Characterisation of Polysaccharides

#### 4.3.1. Molar Mass Distribution

The profile associated with the molar mass distribution of the samples was estimated by High Performance Size Exclusion Chromatography (SEC) using two columns in series: TSKGel G3000PW_XL_ and TSKGel G2500PW_XL_ (300 × 7.8 mm) with a pre-guard column PWX-guard (40 × 6 mm) (Tosoh Bioscience, Griesheim, Germany) performed in a 1100 series Hewlett-Packard chromatograph (Agilent, Waldbronn, Germany). Samples were previously dialysed using membrane tubing (MWCO 0.5 kDa, SpectrumLabs, San Francisco, CA, USA). The detector used was a refractive index, and the mobile phase was Milli-Q water at 0.4 mL/min at 70 °C. The standards used were dextrans from 1000 to 80,000 g/mol (Honeywell Fluka, Charlotte, NC, USA).

#### 4.3.2. Fourier-Transform Infrared Spectroscopy (FTIR)

The characteristic bands associated with different groups were determined by FTIR. The lyophilised extracts were blended with KBr and dried for 30 min using an infrared lamp. The measurements were performed on a Bruker IFS 28 Equinox (Billerica, MA, USA), and the software for data acquisition was OPUS-2.52. The range of the spectra was from 400 to 2000 cm^−1^ (25 scans/min).

#### 4.3.3. Oligosaccharide Contents

The extracts were dissolved in Milli-Q water, hydrolysed (4% sulfuric acid, 121 °C, 20 min), and dialysed (membrane tubing MWCO 0.5 kDa, SpectrumLabs, San Francisco, CA, USA). Monosaccharide contents were determined by high-performance liquid chromatography (HPLC) using a 1100 series Hewlett-Packard chromatograph (Agilent, Waldbronn, Germany) equipped with a refractive index detector and a Aminex HPX-87H column with pre-guard (300 × 7.8 mm, BioRad, Hercules, CA, USA). The separation of the monosaccharides was performed at 60 °C using sulfuric acid (0.003 M) as a mobile phase at 0.6 mL/min. The quantification was expressed as oligossacharide.

### 4.4. Statistical Analyses

For biochemical characterisation and MIC data, statistical analysis was carried out in SPSS version 23.0. Differences in biochemical composition and microbial growth were analysed by ANOVA with Tukey HSD post hoc tests. Growth parameters from bacterial growth curve analysis were determined using DMFit Excel software (version 3.5, ComBase, Wyndmoor, PA, USA). Optical density and incubation time were plotted on DMFit and fitted to the Gompertz model [92], where the goodness of fit was determined by a coefficient of regression (R^2^). The primary growth parameters, rate (µmax) (optical density/30 min interval), and lag (λ) (h) were determined from extrapolation of the model. The correlations between the growth and composition parameters were analysed in R [93] version 4.3.2 (accessed on 4 April 2024), while “ggplot2” and “corrplot” were used to generate the Pearson’s correlation matrix [94] and “cor.mtest” was used to include the *p*-values within the matrix. The variance within the full data set was explored by a principal component analysis (PCA) using Varimax rotation with Kaiser normalisation to extract the eigenvalues values of the components using SPSS version 23.0.

## 5. Conclusions

Extracts of *A. esculenta*, a brown macroalgae common in the North Atlantic, have been shown in this study to possess antimicrobial activity against *E. coli*, one of the most common pathogens relevant for food safety. The authors suggest that the observed antimicrobial activity is due to the quantity of polysaccharides in the extracts, which are known to contribute to the antimicrobial activity of seaweeds. The strongest antimicrobial activity from these extracts was observed from the extract prepared using UAE with an MIC of 6.25 mg/mL. No antimicrobial activity was seen against *L. innocua*. Further polysaccharide analyses of these extracts indicated the presence of fucoidan and laminarin, some of the most widely reported polysaccharides present in brown macroalgae with associated bioactivities. Future research should focus on further fractionation of these extracts to elucidate antimicrobial compounds and further examination of these extracts in an applied setting relevant for food safety. For example, these extracts may be investigated for use as an antimicrobial agent incorporated in the formulation of food products, during the food washing process, or as part of active food packaging (for example incorporated into compostable plastic wrap). In conclusion, this study indicates the potential for extracts of the brown seaweed *A. esculenta* to be used as an antimicrobial agent in food safety settings.

## Figures and Tables

**Table 1:** Microbial growth parameters (rate (µ_max_) and lag (λ)) of *E. coli* DSM1103 incubated with extracts of *A. esculenta* extracted with either microwave-assisted extraction (MAE), ultrasound-assisted extraction (UAE), ultrasound–microwave-assisted extraction (UMAE), and maceration. Rate (µ_max_) and lag (λ), as well as the coefficient of determination (R^2^), were extrapolated from Gompertz modelling. Results of µ_max_ and λ are expressed as average ± standard deviation (n = 4). Each extract was applied at its MIC (12.5 mg/mL for MAE, 6.25 mg/mL for UAE, 12.5 mg/mL for both UMAE and maceration extract). Different letters indicate a significant difference (*p* < 0.05) between means. Abbreviations in the table are as follows: optical density at 600 nm measured every 30 min (OD_600 nm_ 0.5 h^−1^), (h) hours.

Sample	Rate μ_max_ (OD_600 nm_ 0.5 h^−1^)	Lag λ (h)	R^2^
*E. coli* DSM1103 alone	0.08 ± 0.003 ^c^	5.01 ± 0.16 ^a^	0.98
*E. coli* + MAE extract	0.12 ± 0.010 ^b^	5.73 ± 0.17 ^b^	0.99
*E. coli* + UAE extract	0.12 ± 0.012 ^b^	5.15 ± 0.22 ^a^	0.99
*E. coli* + UMAE extract	0.12 ± 0.008 ^b^	5.34 ± 0.04 ^a^	0.99
*E. coli* + Maceration extract	0.15 ± 0.011 ^a^	5.28 ± 0.24 ^a^	0.99

**Table 2:** Monosaccharide composition (g oligosaccharide/100 g dried extract) of extracts generated by either microwave-assisted extraction (MAE), ultrasound-assisted extraction (UAE), ultrasound–microwave-assisted extraction (UMAE), and maceration from the brown seaweed *A. esculenta*. Results are expressed as the average ± standard deviation (n = 3). Different letters indicate a significant difference (*p* < 0.05) between means. The retention time (RT) of compounds was as follows: glucuronic acid (RT 8.29 min), glucose (RT 9.42 min), galactose (RT 10.11 min), rhamnose (RT 10.63 min), and fucose (RT 11.617 min).

Hydrolysed Extract	Glucuronic Acid	Glucose	Galactose	Rhamnose	Fucose
MAE extract	2.25 ± 0.15 ^a^	15.73 ± 1.05 ^c^	2.31 ± 0.38 ^b^	3.66 ± 0.37 ^c^	0.52 ± 0.10 ^c^
UAE extract	3.77 ± 0.35 ^b^	11.07 ± 0.57 ^d^	1.90 ± 0.25 ^b^	4.99 ± 0.37 ^c^	1.28 ± 0.20 ^b^
UMAE extract	2.38 ± 0.11 ^a^	33.27 ± 0.67 ^a^	3.91 ± 0.45 ^a^	7.18 ± 0.80 ^b^	2.60 ± 0.04 ^a^
Maceration extract	4.48 ± 0.44 ^b^	20.28 ± 0.10 ^b^	3.94 ± 0.22 ^a^	12.89 ± 0.48 ^a^	0.66 ± 0.17 ^c^

## Data Availability

Data will be made available upon request.
